# Effect of Magnetopriming on Photosynthetic Performance of Plants

**DOI:** 10.3390/ijms22179353

**Published:** 2021-08-28

**Authors:** Mohammad Sarraf, Kricelle Mosquera Deamici, Houda Taimourya, Monirul Islam, Sunita Kataria, Ritesh Kumar Raipuria, Gholamreza Abdi, Marian Brestic

**Affiliations:** 1 Department of Horticulture Science, Shiraz Branch, Islamic Azad University, Shiraz 71987-74731, Iran; sarraf.science@gmail.com; 2 Faculty of Pharmacy, Federal University of Bahia, Salvador 40170-115, BA, Brazil; kricelledeamici@gmail.com; 3 Department of Horticulture, Horticol Complex of Agadir (CHA), Agronomy and Veterinary Institute Hassan II, Agadir 80000, Morocco; houdataimourya@yahoo.com; 4 Department of Sustainable Crop Production, Università Cattolica Del Sacro Cuore, Via Emilia Parmense 84, 29122 Piacenza, Italy; monirul.islam@unicatt.it; 5 School of Biochemistry, Devi Ahilya Vishwavidyalaya, Khandwa Road, Indore 452001, India; 6 ICAR-National Institute for Plant Biotechnology, New Delhi 110012, India; raipuriaritesh@gmail.com; 7 Department of Biotechnology, Persian Gulf Research Institute, Persian Gulf University, Bushehr 7516913817, Iran; abdi@pgu.ac.ir; 8 Department of Plant Physiology, Slovak University of Agriculture, Nitra, Tr. A. Hlinku 2, 949 01 Nitra, Slovakia; 9 Department of Botany and Plant Physiology, Faculty of Agrobiology, Food, and Natural Resources, Czech University of Life Sciences Prague, Kamycka 129, 165 00 Prague, Czech Republic

**Keywords:** biomass, leaf growth, magnetopriming, photosynthetic performance, photosynthetic enzymes, PSII efficiency

## Abstract

Magnetopriming has emerged as a promising seed-priming method, improving seed vigor, plant performance and productivity under both normal and stressed conditions. Various recent reports have demonstrated that improved photosynthesis can lead to higher biomass accumulation and overall crop yield. The major focus of the present review is magnetopriming-based, improved growth parameters, which ultimately favor increased photosynthetic performance. The plants originating from magnetoprimed seeds showed increased plant height, leaf area, fresh weight, thick midrib and minor veins. Similarly, chlorophyll and carotenoid contents, efficiency of PSII, quantum yield of electron transport, stomatal conductance, and activities of carbonic anhydrase (CA), Rubisco and PEP-carboxylase enzymes are enhanced with magnetopriming of the seeds. In addition, a higher fluorescence yield at the J-I-P phase in polyphasic chlorophyll a fluorescence (OJIP) transient curves was observed in plants originating from magnetoprimed seeds. Here, we have presented an overview of available studies supporting the magnetopriming-based improvement of various parameters determining the photosynthetic performance of crop plants, which consequently increases crop yield. Additionally, we suggest the need for more in-depth molecular analysis in the future to shed light upon hidden regulatory mechanisms involved in magnetopriming-based, improved photosynthetic performance.

## 1. Introduction

Photosynthesis is the process that makes plants diverse organisms on Earth. The primary function of photosynthesis is to convert light energy into chemical energy, which is the key function in plant life and the food chain for animals, but this can be influenced by many environmental factors. Several studies have shown that the photosynthetic process can be affected by high or low light intensity, high or low temperature, heat, salinity, drought, UV-B stress, electrical signals, and geomagnetic field intensity [1,2,3,4,5,6,7,8]. The main characteristics of photosynthetic damage indicate lower activity of photosynthetic enzymes, decreased assimilation of carbon dioxide (CO_2_), quantum yield of photosystem II (ΦPSII) and increased nonphotochemical quenching (NPQ) [6,7,9,10,11]. However, photosynthetic light reactions are actively related to the transport of electrons through the chloroplast electron transport chain to ion fluxes of thylakoid membranes, which are particularly charge transferred [12,13,14].

Among the natural components of our planet, such as water, temperature, climate and electric charges, Earth’s magnetic field or geomagnetic field (GMF) is a component that influences many biological processes in plants [15,16,17,18]. As a sessile organism, plants show different levels of morphophysiological and molecular responses under different magnetic field (MF) intensities, such as shoot, root and stem elongation, photosynthetic performance, plant nutrient uptake and the expression of several genes associated with photoreceptors [8,18,19,20]. Accordingly, many researchers have used a static magnetic field (SMF) to influence plant growth and development and to reduce cellular oxidative stress under unfavorable environmental conditions. At the present state, for sustainable agriculture, researchers are looking for new environmentally friendly approaches that can contribute to increasing crop yield, but at the same time, they must have a low ecological impact.

In this sense, magnetopriming (exposure of seeds to a MF) is a simple, efficient method having significant worth because it can mitigate abiotic or biotic stress. Various reports have proven that magnetopriming improves seed germination, plant growth, physiology, antioxidant activity, photosynthetic performance and yield under different abiotic stresses, such as drought, salt, UV-B, and arsenic stress [21,22,23,24].

**Figure 1:**
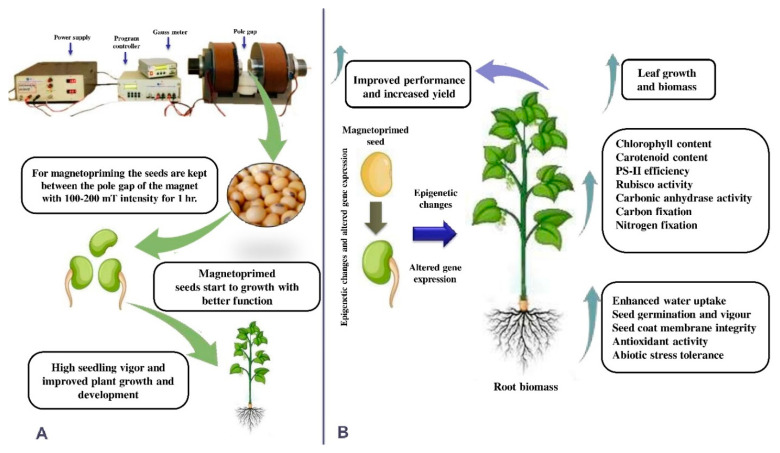
(**A**) Steps of magnetopriming from seed to plant maturity; (**B**) various effects of magnetopriming from seed germination to plant maturity contribute to enhance photosynthetic performance under nonstress and stressed conditions.

Figure 1 illustrates a seed treatment with a magnetic field and various effects of magnetopriming persisting from seed germination to plant maturity. The combined effect of these improved parameters enhances plant growth, biomass, photosynthesis, and yield under nonstress and stressful conditions.

However, many other studies have reported that SMF treatment enhances photosystem II (PSII) efficiency, photosynthetic pigments (chlorophyll *a* and *b*), and the performance index, as well as leaf gas exchange performance [19,22,23,25,26,27]. Hence, the present review aims to present different MF applications and their effects on photosynthetic performance for sustainable agriculture systems.

## 2. Effect of a MF on Photosynthetic Pigments

Exposure to a MF could be useful to enhance plant growth and productivity in addition to overall biomass production, and improvement also affected metabolic substances such as plant photosynthetic pigments. In fact, this better photostimulation and growth can be explained by improving ion uptake and mobilization under a MF [28]. In contrast, plant growth and productivity are generally controlled depending on photosynthetic pigments [29]. Indeed, MFs are known to promote biochemical changes and could be used as a tool to stimulate growth and responses, including photosynthetic pigments such as chlorophyll and carotenoids [30]. A SMF showed a simulative effect on pigment content (carotenoids, chlorophyll *a*, *b*, and total pigments), whereas carotenoids and chlorophyll *a* were more affected than chlorophyll *b* [19]. Chlorophylls are vital pigments that absorb a considerable amount of light energy and perform photosynthetic reactions in plants. Another observation showed that prolonged MF exposure time of a SMF (100 mT for 360 min) treatment significantly increased the level of photosynthetic pigments in date palm [31]. Thus, photosynthetic pigment content showed a considerable enhancement in response to a MF at low doses.

The photosynthetic pigments (chlorophyll *a* and *b*) increased obviously in strawberry and tomato plants cultivated in magnetically treated culture medium compared with those cultivated in normal nutrient solution. The increases in chlorophyll a content in strawberry and tomato plants were 345.4% and 99.1% compared with the controls, respectively. The percentage of chlorophyll *b* content in strawberry and tomato plants increased by 255.9% and 108.4% compared with the controls, respectively [32].

This is in agreement with earlier findings in a similar experiment under greenhouse conditions, where photosynthesis and chlorophyll content of maize plants increased from magnetically exposed seeds to a SMF of 100 mT for two hours and 200 mT for 1 h, when compared with untreated seeds, under water stress [33]. These results agree with those of Abdul Qados and Hozayn [34], who found increases of 17.46% and 67.8% in chlorophyll *a* and chlorophyll *b* contents in flax plants, respectively. Moreover, Baghel et al. [27] reported that an enhancement of 126% in total chlorophyll was recorded in plants that emerged after a 200 mT SMF treatment of soybean seeds compared with the untreated control. Even under salt stress conditions, this enhancement reached 58% at 50 mM salinity for plants obtained from SMF-treated seeds compared with untreated seeds.

**Figure 2:**
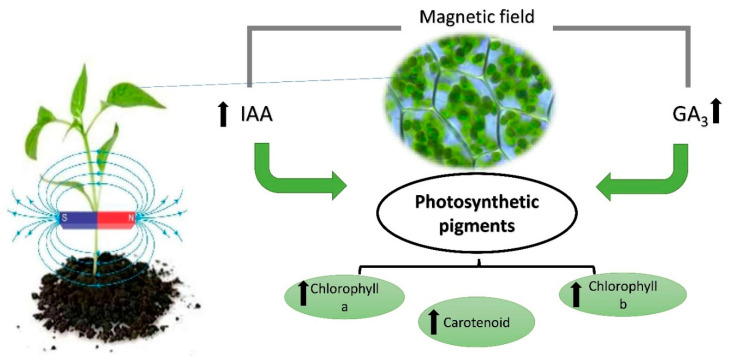
The performance of photosynthetic pigments under the influence of a magnetic field.

The increases in photosynthetic pigment content in response to a MF were confirmed by several studies for different plants: broad bean, chickpea, tomato, date palm, common bean, sunflower, potato and sugar beet [25,35,36,37,38,39,40,41]. These significant increases in photosynthetic pigment contents may be attributed to the enhancement in growth promoters (indole acetic acid (IAA)), which increased protein contents [34,36]. In this context, Çelik et al. [42] found a stimulatory effect on photosynthetic pigments as a result of the beneficial effect of a MF on protein synthesis. In addition, Atak et al. [43] explained the increases in all photosynthetic pigments through the increase in cytokine synthesis induced by a MF. In addition, El Sayed [36] found that irrigation of broad bean plants with magnetically treated nutrient solution (MTNS) significantly increased the gibberellic acid (GA3) and kinetin contents compared with the control. A general overview of MFs and their function in photosynthetic pigments is shown in Figure 2.

The specific effects of the application of a MF on carotenoids and chlorophyll (*a,b*) and total chlorophyll (a+b) content have been stated for different plant species such as sugar beet, sunflower, soybean, maize, and mung bean under nonstress conditions [25,44,45,46], as well as in the presence of salt, water, UV-B and cadmium toxicity [8,21,22,27,47,48,49,50]. Likewise, under drought stress and nonstress conditions, pre-sowing electromagnetic treatments caused improvements in chlorophyll (*a* and *b*) contents [51]. The MF treatment-induced enhancement in chlorophyll pigments may possibly be due to the presence of paramagnetic properties of chloroplasts, which may be capable of supporting the rate of seed metabolism [25,52]. Other possible explanations for the increase in pigments are that the magnetic moments of the atoms in MFs are affected and oriented downwards in the field direction. Given that chloroplasts have paramagnetic properties [53], the influence of a MF on plants increases its inner power, which is distributed among the atoms, accelerating plant metabolism [53].

Similarly, carotenoids help plants absorb light energy for use in photosynthesis, since this pigment protects the plants by scavenging reactive oxygen [54], which is known to be affected by magnetic treatment [55]. Conversely, it has been reported that longer exposure decreased the level of photosynthetic pigments in *Zea mays* L. and *Robinia pseudoacacia* L. seedlings [56,57]. These decreases were linked to the effect of the MF on the reduction in plastids inside the cells [58]. Using MF treatment could be a promising technique for agricultural improvements, but extensive research is required, using different levels of MF doses to determine the optimum dose.

## 3. Effect of MF on Chlorophyll Fluorescence

The kinetic analysis of chlorophyll *a* fluorescence (Chl F) has become an important tool in basic research on agronomy and plant physiology, representing a new approach to studying the photosynthetic performance of leaves under nonstress and abiotic stresses. The analysis of fluorescence signals is a simple, fast and sensitive method to monitor the changing physiological states of the photosynthetic system [59] that provides accurate information on the status and PSII function and light-harvesting antenna complexes in addition to the transferor and acceptor sides of PSII [12].

**Figure 3:**
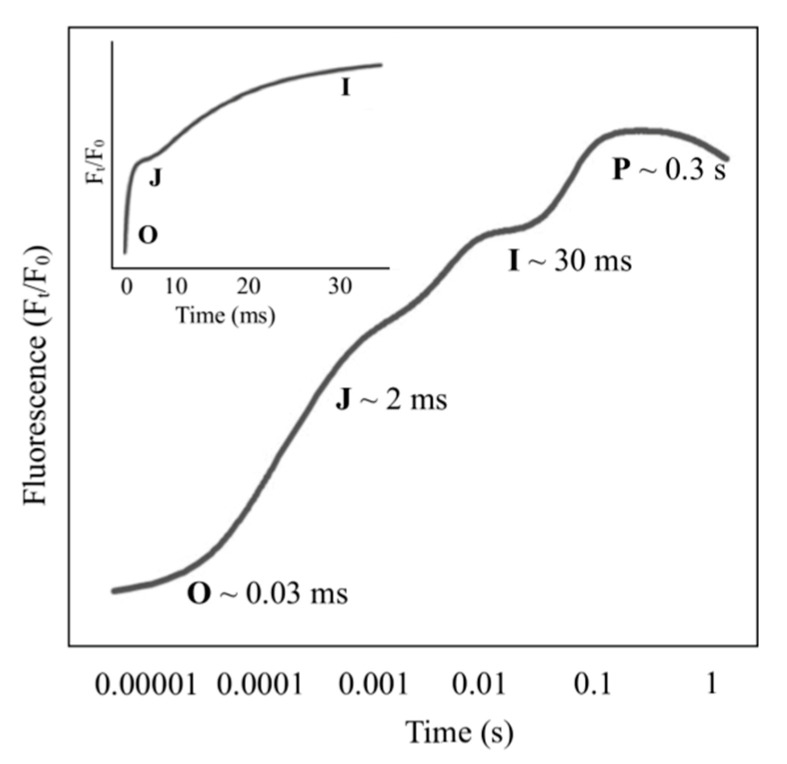
Typical chlorophyll *a* polyphasic fluorescence curve exhibited by plants by the transient plotted on a logarithmic time scale. The curve plotted on a regular time scale is shown at the bottom, modified from Kalaji et al. [12].

Typically, the fluorescence rise in dark-adapted intact leaves after illumination with high actinic light intensity plotted on a logarithmic time scale displaying a polyphasic chlorophyll fluorescence induction curve: O, J, I and P phases (Figure 3). The trajectory of the OJIP curve is a specific point on the induction curve formed by the recorded Chl F signal [12] and provides some information regarding the functions, conformation and structure of the photosynthetic apparatus [59,60]. The JIP test (OJIP) corresponds to the gradual reduction of QA and the primary electron acceptor of PSII [12], and the shape of this curve depends on PSII grouping (L-band) [61] and the balance between electron donation from the oxygen-evolving complex (OEC) to the excited PSII reaction center (P680+) and electron acceptance from the QA-(K-band) [61]. The general behavior of the OJIP curve corresponds to an initial fluorescence Fo (phase O), where the fluorescence transient starts. Then, there are two intermediate steps, FJ and FI (phases J and I, respectively), before it reaches the maximum FM (phase P) [62]. The O-J part corresponds to the closing of some PSII reaction centers due to the reduction of QA to a level between the trapping rate and QA reoxidation rate by QB and the other part of the electron transfer chain. J-I corresponds to the reduction of plastoquinone (PQ), cytochrome (Cyt b6f), PC and the secondary electron acceptor QB. The rise in the I-P part is usually attributed to the reduction of some electron transporters, such as ferredoxin, intermediary acceptors and NADP, from the PSI acceptor side [12].

Chl fluorescence analysis also gives an important parameter, the quantum yield, represented by the FV/FM ratio, which is used as the main indicator to evaluate PSII performance [63]. FV corresponds to the variable fluorescence, calculated by the difference between the maximum fluorescence (FM) and minimal fluorescence (F0) [64].

In the case of plant photosynthesis, the observed MF-stimulating effects have been described in regard to the evolution of a radical pair appearing in PSII by Voznyak et al. [65]. The study reported by these authors showed that the MF stimulated fluorescence changes in PSI. The experiments were performed on P-700-enriched complexes isolated from pea chloroplasts. MF-stimulated effects in photosynthetic algae and bacteria were explained by a hypothesis of radical pair recombination in reaction centers [65].

**Figure 4:**
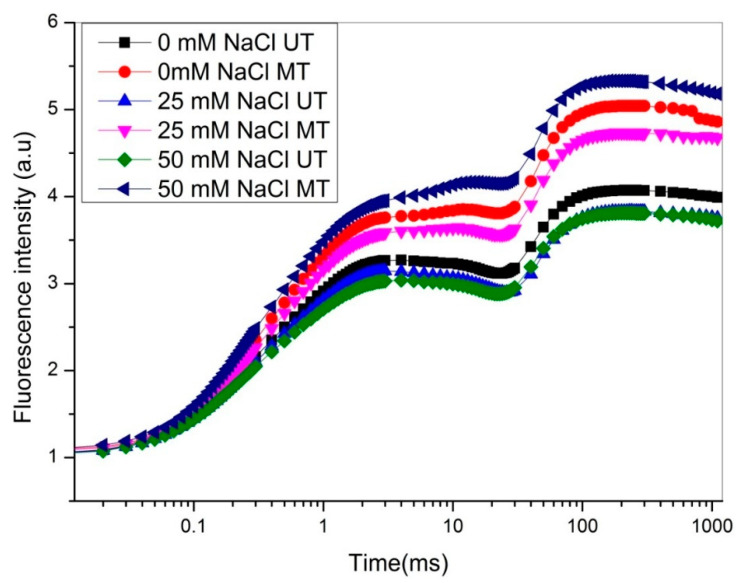
Effect of SMF pretreatment on the typical chlorophyll-a polyphasic fluorescence curve exhibited by third trifoliate leaves of soybean plants under salt stress (25 and 50 mM NaCl) by transient plotting on a logarithmic time scale. Modified from Baghel et al. [27]. UT = the plants that emerged from untreated seeds, and MT = the plants that emerged from SMF-pretreated seeds.

The effect of SMF 200 mT for one hour on polyphasic Chl F transient was studied in soybean trifoliate leaves to evaluate the photochemical efficiency of PSII under nonstress and abiotic stress conditions, such as water, salt, UV-B, and heavy metal toxicity [8,19,21,22,23,24,27,49,50]. The results of these studies indicated the positive effects of SMF pretreatment on plant growth, photosynthesis, nitrogen metabolism, performance index, PSII efficiency, and yield under nonstress and stressed conditions [8,19,21,22,23,24,27,49,50]. Electromagnetic treatment was applied at strengths of 100 and 150 mT for 10 min to corn seeds, which mitigated the drought-induced adverse effects on growth through the improvement of PSII efficiency and other parameters [51]. Relating to the fluorescence yield in dark-adapted trifoliate leaves, the time course plotted on a logarithmic time scale illustrates that the separation of OJIP phases with SMF treatment showed a higher fluorescence yield at the I and P phases when the plants were grown under nonstress and abiotic stress conditions (water, salt, UV-B and heavy-metal) compared with the plants obtained from untreated seeds [8,19,21,22,24,27,49]. Figure 4 shows the higher fluorescence yield at the I and P phases in the third trifoliate leaves of soybean plants that were obtained from SMF-treated seeds (MT) compared with the leaves of plants from untreated (UT) seeds grown under salt stress (0, 25, and 50 mM NaCl) [27]. The rise in the fluorescence curve after SMF treatment was due to the result of a faster decrease in electron acceptors in the photosynthetic pathway downstream of PSII, particularly Q_A_ and plastoquinone [19]. The results of these studies concluded that SMF pretreatment enhanced the tolerance of plants to abiotic stress conditions. SMF pretreatment could ameliorate the inhibition of growth, OJIP test parameters and PSII efficiency as a result of supplemental and ambient UV-B stress in plants [8,49,50]. These authors showed that ambient and supplemental or enhanced UV-B stress caused a reduction in the I-P phase of the OJIP curve in third trifoliate leaves of plants that emerged from untreated seeds, while plants that grew from SMF-treated seeds revealed a noteworthy enhancement in the I-P phase under UV-B stress. The IP phase is correlated with electron transfer through PS I [66]. Several performance indices (PIs) have been identified that provide information on the efficiencies of specific electron transport reactions in the thylakoid membrane and the structure and function of PSII [67]. Kataria et al. [8] observed that Fv/Fm, the maximum quantum yield (efficiency) of PSII, ΔV (I-P phase, the amplitude of the comparative contribution of the I-to-P rise for the OJIP transient), φEo, the quantum yield of electron transport, PIABS, performance index at absorption basis and PItotal, total performance index were significantly further improved by SMF-pretreatment than generally used parameters Fv/Fm under ambient UV-B and supplemental UV-B stress, and it was found to be well connected with photosynthetic capability measured as assimilation of CO_2_ [68].

Thus, it has been suggested that higher OJIP-test parameters, such as Fv/Fm, Fv/Fo, φEo = ETo/ABS, ∆V(IP), PI total and PIABS, in the plants that emerged after SMF treatment contribute to higher light-harvesting efficiency, and as a result, it caused an increase in the biomass accumulation and uptake of CO_2_ under nonstress and abiotic stresses, such as water, salt, and UV-B stress, thereby enhancing all yield parameters of the crop plants [8,21,22,27,49,50,69]. Furthermore, Chl *a* fluorescence studies revealed that leaves of SMF-treated plants have higher reducing power with more active reaction centers and higher efficacy of electron transport than untreated plants under nonstress conditions and in the presence of water, salt and ambient UV-B stress [19,21,22,44,49,50,69].

The stress conditions that plants are exposed to are responsible for alterations in their physiology, morphology, physiology and biochemistry, which negatively or positively affect their growth and productivity. Considering the significant effect of MF treatment, particular conditions of time exposure and intensity could cause different effects on the photosynthetic apparatus and Chl *a* analysis.

## 4. Effect of MFs on Photosynthesis

Photosynthesis provides the basis for life on Earth by removing CO_2_ from the atmosphere and releasing oxygen [70]. It consists of a physical-chemical process of converting CO_2_ and sunlight into energy and organic matter [71,72]. This process can be defined as a reduction reaction based on the light energy captured by the chlorophyll molecules present in plants in which CO_2_ and water are converted into carbohydrates and oxygen. Photosynthesis is divided into a light phase that takes place in the thylakoid and a dark phase that occurs in the stroma, and both take place in chloroplasts. Light-dependent reactions consist of two main steps, carried out by two main photoactive complexes, PSI and PSII, that carry out electron transport and interact with each other indirectly through a chain electron carrier. Photosynthesis starts at the PSII complex by capturing sunlight, and then electrons transfer to PSI, which are oxidized by light, reducing NADP+ to NADPH and ferredoxin, which are further used in CO_2_ fixation reactions in the Calvin cycle, also known as the dark phase [73,74,75].

**Figure 5:**
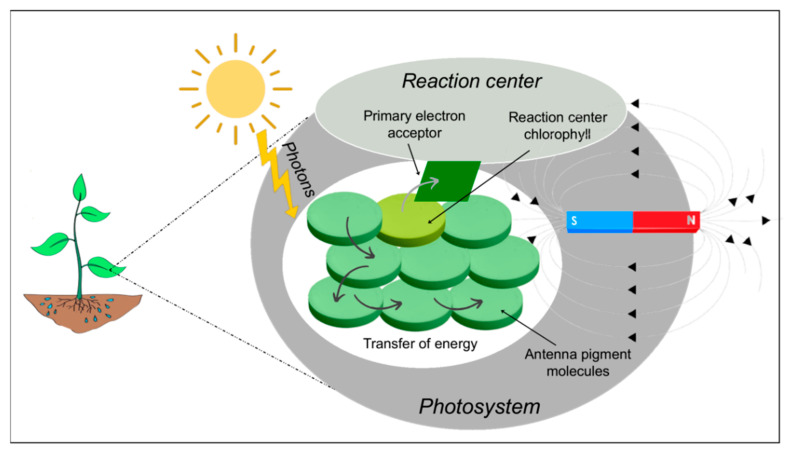
Magnetic field action on the plant photosynthetic system.

The photosynthetic process is a very important parameter of plant metabolism that can be used to evaluate the health status of plants since plants are usually very sensitive to environmental changes. Figure 5 represents the plant photosynthetic system with MF action. Numerous authors have investigated the effects of MFs on the metabolism and growth of microalgae and several plant species [8,27,50,63,76,77]. The first study on MF effects on plants was conducted by Krylov and Tarakonova [78], and currently MFs are studied as a pretreatment in agriculture for seed priming, aiming to improve seed germination, growth and photosynthesis [8,23,49,50,69,79,80]. Photosynthetic organisms, such as cyanobacteria, algae and plants, are fundamental to life on Earth because of the conversion of solar energy, water and CO_2_ to chemical energy [71]. To date, several studies have been carried out to evaluate the response of various plant species under different ranges of MF intensities. Among these studies, Pittman [81] observed that a MF of relatively low intensity may possibly be effective in stimulating or initiating plant growth responses, and afterward, other studies showed different effects also with high intensities.

Shine et al. [19] investigated the effect of a SMF of 0–300 mT on soybean seeds for 30, 60 and 90 min. The results demonstrated that a MF increased germination-related parameters, such as speed of germination, water uptake, seedling length, biomass accumulation and vigor indices. As a more effective treatment, MF application at 200 and 150 mT for one hour promoted growth, leaf protein content and photosynthetic efficiency. Anand et al. [33] evaluated the effects of a SMF on maize plants in a similar experiment under field conditions and showed that a SMF of 200 mT for one hour and 100 mT for two hours was sufficient to increase photosynthesis and Chl content when the maize plants were compared with the control under irrigated and mild-stress conditions. Other studies have demonstrated that SMF pretreatment causes an increase in the rate of photosynthesis and stomatal conductance [8,22,49,69,82,83], as well as the biomass accumulation in crop plants under abiotic stresses, such as salt, water, arsenic and cadmium toxicity and ambient and enhanced UV-B stress [8,21,22,24,27,48,49,50]. Thus, pre-sowing SMF treatment can be effectively used to alleviate the adverse effects of abiotic stress in crop plants by increasing the photosynthetic performance of the plants.

## 5. Effect of MFs on Photosynthetic Enzymes

The photosynthetic performance of any plant is also dependent on the activity of photosynthetic enzymes, such as CA, Rubisco, and PEP carboxylase, along with the efficiency of PSI and PSII. It is a well-known fact that efficient photosynthesis leads to higher biomass accumulation, which primarily determines overall plant performance, including yield, under normal and stressed conditions [84]. Seed priming with MF treatment has been proven to improve the photosynthetic performance of various crop species through increased efficiency of PSI, PSII, OJIP test parameters and gas exchange parameters in nonstress and abiotic stress conditions [8,15,21,22,23,27,33,49,69]. Whether this improved photosynthetic performance of plants by magnetopriming is due to improved activities of enzymes related to photosynthesis has not been explored much, but a few reports are available as a proof-of-concept in this regard. Total protein gel profiling of trifoliate leaves originating from unprimed and SMF-primed soybean seeds was performed by Shine et al. [19]. The SDS gel profile showed greater band intensities of the Rubisco large subunit (53 kDa) and small subunit (14 kDa) in SMF-treated samples than in untreated samples [19]. Similarly, Patel [85] also found that the expression of genes related to the Rubisco large subunit, PEP carboxylase and CA enzymes was higher in the leaves of plants from SMF-primed seeds compared with unprimed seeds. The CA enzyme is known to be involved in the first step of C_4_ photosynthesis, which is the conversion of CO_2_ molecules to HCO_3_ via hydration. Recently, it has been shown that SMF priming of soybean and maize seeds enhanced the CA activity in the leaves under nonstress and ambient UV-B stress conditions with respect to their unprimed control plants, which helped alleviate the detrimental effects of ambient UV-B stress [50]. Likewise, the activity of CA and PEP carboxylase activity were significantly increased by magnetopriming under nonstress conditions in maize plants [85]. Thus, the results from the literature suggest that the linear flow of electrons beyond PSII may be increased by magnetopriming, as evident by the Chl *a* fluorescence data, which leads to a greater proportion of electrons available for the Calvin cycle, it may be accountable for magnetopriming enhanced expression and activity of photosynthetic enzymes such as Rubisco, CA and PEP-carboxylase.

## 6. Effects of Magnetic Field Treatment on Leaf Features

Leaf morphological features (leaf length, width, area, shape, and venation network) and anatomical features (mesophyll cell and bundle sheath cell organization and vasculature) determine the quantity of light interception and photosynthetic capacity [86]. Similarly, leaf venation provides mechanical support along with mobilization of photosynthates from source to sink [86,87]. Whether magnetopriming of seeds alters leaf features to improve photosynthesis has not been explored. In this regard, synchrotron-based, phase-sensitive imaging revealed that magnetopriming of soybean seeds (200 mT for 1 h) increased the thickness of the midrib, minor veins and area of third trifoliate leaves compared with unprimed leaves [24,87,88]. Furthermore, midrib thickness and area enhancement led to an increased rate of water uptake, photosynthesis, and stomatal conductance. These altered features of leaves by magnetopriming have not been explored much, and plant researchers working in this area should explore these opportunities, which will further improve our understanding regarding the mechanisms involved in magnetopriming-induced improvement of crop photosynthetic performance.

## 7. Effect of MFs on the Yield of Plants

The beneficial effect of magnetic treatment on plant yield has been widely demonstrated in many plant species (Table 1). In spring maize, incremental effects of magnetic field treatment of water have been noticed in plant yield [89]. Seed magnetopriming not only alleviated salt stress effects but also resulted in an outstanding boost in yield attributes in saline and non-saline conditions [22,27]. Seed exposure to a SMF has the potential to increase crop production per unit area of land without having any negative effects on any environmental component [22,27,69]. Static and pulsed MF treatment was found to increase the productivity of cherry tomatoes under a controlled environment [90]. The magnetopriming of soybean seeds allowed SMF-treated plants to overcome the harmful effects of water, salt, and UV-B stress on growth, biomass accumulation and yield in terms of the number of seeds and pods per plant, weight of seeds and pods per plant, and harvest index [21,27,49,69].

## 8. Conclusions and Future Perspectives

Photosynthesis is considered a major determinant of crop productivity. Globally, the plant scientific community is continuously making efforts toward improving the photosynthetic performance of crop species. Genetic manipulations of various processes of photosynthesis alone cannot bring a drastic increase in crop yield in an unpredictable changing global environment. Magnetopriming of seeds has emerged as one of the simplest, efficient, noninvasive methods to improve seed vigor. Magnetoprimed seeds germinate faster and perform well under normal and stressed conditions. In this review, we have discussed the effects of magnetopriming on various photosynthetic parameters of plants. Photosynthesis is a multistep process, and the effect of magnetopriming on these steps has not been explored much thus far. However, magnetopriming-induced increases in photosynthetic pigments, J-I-P test parameters, activities of photosynthetic enzymes, biomass accumulation and rate of photosynthesis and stomatal conductance are evident from the literature. Leaf architectural features dominantly regulate the photosynthetic efficiency of plants. These features are affected by magnetopriming and could not be explored thoroughly. This has the potential to increase crop production through higher photosynthetic activities even under abiotic stress conditions without having any damaging effect on environmental components. Currently, whole transcriptome, proteome and metabolome analyses have become easy to perform, which could provide an idea about the genes and proteins that come into play after magnetopriming and improve the photosynthetic performance of plants. The global population is growing rapidly, and crop yield should also be increased proportionally to match the demand for food. In this scenario, magnetopriming is an easy option to improve crop performance and yield even under stress conditions; however, there is still a need for further research on the effect of magnetopriming on photosynthetic performance under biotic stresses, and detailed studies are required on the effect of MFs on photosynthetic enzyme activities. Additionally, detailed studies on magnetopriming-induced molecular signatures will pave the way to exploit this technology for improved crop performance globally.

## Figures and Tables

**Table 1:** A summary of the effect of magnetic fields on plants to alleviate different abiotic stresses.

MF Optimal Intensity	Stress Condition	Plant Species	Effects	References
200 mT	Water stress	*Glycine max* L.	Increased the plant growth attributes, photosynthetic performance, biomass accumulation, and crop yield	[21]
200 mT	Salinity stress	*Glycine max* L.	An enhancement of growth attributes, photosynthetic performance and crop yield	[27]
200 mT	Salinity stress	*Zea mays* L.	Enhanced seedling vigor growth parameters, PSII photochemistry (Fv/Fm) and crop yield	[22]
200 mT	Arsenic (As) toxicity tolerance	*Glycine max* L.	Reduced As toxicity and increased plant growth parameters with noticeable increase in water uptake, stomatal conductance, PSII performance and photosynthesis	[24]
200 mT	Ultraviolet-B radiation tolerant	*Glycine max* L.	Increased photosynthetic performance along with higher crop yield, decreased H_2_O_2_ content and antioxidant levels	[8]
200 mT	Ultraviolet-B radiation tolerant	*Glycine max* L.	Significant enhancement in growth parameters and higher expression of genes related to amylase, NR and NOS enzymes	[91]
50 mT	Salinity stress	*Triticum aestivum* L.	Increased total chlorophyll contents and Na^+^/K^+^ ratio and growth attributes	[84]
100 mT	Salinity stress	*Cicer arietinum* L.	Enhanced physiological traits, antioxidant activity and Na^+^/K^+^ ratio, overall growth attributes	[92]
136 mT	Drought stress	*Pisum sativum* L. *Apium graveolens* L.	Increased yield and physiological parameters along with Na^+^/K^+^ ratio	[93]
150 mT	Drought stress	*Zea mays* L.	An enhancement of chlorophyll *a* and *b* pigments, leaf water potential, stomatal conductance and decreased total proline contents	[51]
